# Evidence for Emulation in Chimpanzees in Social Settings Using the Floating Peanut Task

**DOI:** 10.1371/journal.pone.0010544

**Published:** 2010-05-12

**Authors:** Claudio Tennie, Josep Call, Michael Tomasello

**Affiliations:** Department of Developmental and Comparative Psychology, Max Planck Institute for Evolutionary Anthropology, Leipzig, Germany; Università di Parma, Italy

## Abstract

**Background:**

It is still unclear which observational learning mechanisms underlie the transmission of difficult problem-solving skills in chimpanzees. In particular, two different mechanisms have been proposed: imitation and emulation. Previous studies have largely failed to control for social factors when these mechanisms were targeted.

**Methods:**

In an attempt to resolve the existing discrepancies, we adopted the ‘floating peanut task’, in which subjects need to spit water into a tube until it is sufficiently full for floating peanuts to be grasped. In a previous study only a few chimpanzees were able to invent the necessary solution (and they either did so in their first trials or never). Here we compared success levels in baseline tests with two experimental conditions that followed: 1) A full model condition to test whether social demonstrations would be effective, and 2) A social emulation control condition, in which a human experimenter poured water from a bottle into the tube, to test whether results information alone (present in both experimental conditions) would also induce successes. Crucially, we controlled for social factors in both experimental conditions. Both types of demonstrations significantly increased successful spitting, with no differences between demonstration types. We also found that younger subjects were more likely to succeed than older ones. Our analysis showed that mere order effects could not explain our results.

**Conclusion:**

The full demonstration condition (which potentially offers additional information to observers, in the form of actions), induced no more successes than the emulation condition. Hence, emulation learning could explain the success in both conditions. This finding has broad implications for the interpretation of chimpanzee traditions, for which emulation learning may perhaps suffice.

## Introduction

The cumulative nature of human culture appears to be unique within the animal kingdom [1]–[5]. This quality requires a high level of copying fidelity to every stage involved; it has been suggested that cumulative culture requires individuals to rely on imitation learning as this leads to learning not only the products, but also the detailed actions necessary to acquire a certain behaviour (i.e., the process leading to the product [5]). Academics remain undecided as to whether non-enculturated (i.e., untrained in any way) chimpanzees learn socially in a comparable way to humans, with some arguing that these chimpanzees engage in imitative learning (e.g. [6]–[8]), and others remaining more sceptical (e.g. [4], [5], [9]). And it is these un-enculturated chimpanzees which represent more closely the state of wild living chimpanzees, since wild-living apes do not have the option of human raising or training (thus, if ecological validity is sought, non-enculturated chimpanzees should be studied). The question of whether or not such chimpanzees can socially learn from others using imitation remains an important debate as it may shed light on what sets human culture apart from other types of cultures in non-human species [4], [5], [9].

Imitation is considered a complex form of social learning that involves copying the demonstrator's bodily actions [10]–[12]. An alternative form of social learning hypothesized to underlie ape traditions is emulation learning ([13], see also [14]). When emulation takes place, the observer “picks up” on changes in the environment that result from the demonstrator's actions, hence the term “results copying” may also be used to describe emulation learning [10], [11]. These results might be perceived and computed in different ways, ranging from so-called “object-movement re-enactment” [15] to insight learning (for a general overview see [16]). An emulator ignores the actions of the demonstrator, and focuses primarily (if not solely) on the changes in the environment. As a consequence, if anything is copied following a demonstration, it will be the results, but not the actions involved.

It is, however, possible that observers are not completely blind to actions insofar as these actions can transmit information about the demonstrator's goals. Observers may therefore learn something about the demonstrator's goals based on the observed actions and in combination with what they understand about the observed results, determine how to achieve the same results (or, if the demonstration failed, they may achieve the opposite result instead; see [17]). The specific details of the actions would however still be lost since focus would be placed on the goals of the demonstrator and not on the actions themselves. Because these goals typically (though not always) revolve around changes in the environment, emulation learning will be required as well. In these cases, the resulting learning type may represent a mixture of goal copying and results copying (named differently by different researchers; e.g., “goal emulation”–with emphasis on the goal copying part [18], but see also [10]; “teleological emulation”–which weighs both learning mechanisms equally [19]).

Empirically, it has proven difficult to separate the effects of actions and results because frequently the two are presented simultaneously (and this is especially problematic if both are somewhat redundant; e.g., a finger pressing a recessed button; see also [20]). For example, Whiten et al. claimed chimpanzees imitated the actions performed by demonstrators during a so-called two-action foraging task [21]. The apparatus in their study had a block obstructing a food chute, which could be moved to release a piece of food by either lifting or pushing the block. In one experimental group, a chimpanzee demonstrator lifted the block by levering an attached bar with a stick, and in another experimental group, another demonstrator used the same stick to push through a hole at the block itself (two different demonstrations–hence *two*-action task). The concern with defining this as imitation, is that if a demonstrator pokes a stick into a hole and the accompanying results (possibly together with the goal of inserting a stick) are copied by an observer, then the observer's behaviour will appear as if they had also copied the underlying actions (the “human eye” seems prone to this type of error). Because of the redundant actions, this experimental design does not rule out emulation as the underlying learning force (which is why it would be more precise to call this method the “two-actions/two-results task”). This kind of methodological issue is not unavoidable, as evidenced by studies on birds (e.g. [22]), dogs [23] and marmosets [24] which overcame these problems by introducing action style components into demonstrations. For great apes, however, most observational learning studies which found copying are unable to distinguish between imitation and emulation learning, because it is unclear exactly what element has been copied (e.g. [21], [25]–[27]). To complicate matters, some such studies often add further potential information types, such as local or stimulus enhancement (e.g. [21], [28]), which again increase the number of possible underlying learning mechanisms (e.g., the two locations in [21]). In addition to the typical confound of mixing action with results information (see above), two-action tasks often involve the introduction of relatively trivial differences between groups (e.g., move a lever to the left or right), which can hardly be regarded as a full blown culture even if the respective methods spread (e.g. [12], [29]). If such traditions are induced they can at best be described as mere “founder effects” of binary types of information/traditions (compare [5]), which (even though important in their own rights) may not get at the heart of the question of whether ape traditions have much in common with human culture. Therefore, in an attempt to answer these questions, less trivial tasks should be used (compare also [17]).

In order to truly investigate whether great apes copy actions spontaneously, a study needs to do one of three things: 1) demonstrate pure actions without any results information at all (“esture copying studies” see [30]); 2) demonstrate pure results without any action information at all (“host control studies” see [12], [29], [31]) or 3) decouple actions and results (this study). In the latter case, (non-redundant) demonstrated actions Y would lead to the result X (e.g., an approach to a hole walking on one's hands and the insertion of the stick using the foot). Due to them being decoupled from the resulting effect, the peculiar actions Y would later only materialize in the observers if they were indeed copying actions. This would then be a direct test of action copying. The logical counter-variant of such studies–used here–may directly test for emulation learning instead. Here, the demonstrator demonstrates the action Y, but, crucially, the setup is such that observers can only perform an unobserved action Z (because action Y is blocked/unavailable to observers). Again, both actions do lead to the same result (X). Here, if observers produce action Z, then action copying cannot have been responsible–because the observers never have seen action Z being demonstrated. Thus, the observers must have used different types of learning (i.e., emulation learning: reproducing the same result–by necessarily re-inventing an unseen action).

There is only one published study to date that uses the “esture copying”method in non-enculturated chimpanzees (i.e., to demonstrate pure actions without results). In this study, chimpanzees failed to copy a novel action (a “begging gesture” from a conspecific model despite potentially high levels of rewards [30]. Although these findings provided no evidence that chimpanzees copy actions spontaneously in problem solving tasks, subsequent ghost control studies have produced a more ambiguous picture. Three ghost control studies with chimpanzees have now been published, all using conspecific-demonstrator conditions as comparisons (i.e. [12], [29], [31]). In one study, observers showed no evidence of observational learning regardless of the condition [12]. In a different study, chimpanzees learned in the full-demonstration condition, but did not learn in the ghost condition [31]. However, in the third study, there was evidence for observational learning in both conditions (i.e., evidence also for emulation in the ghost condition), but with stronger observational learning in the full demonstration condition [29]. In sum, non-enculturated chimpanzees (henceforth simply chimpanzees) do not seem to copy pure actions without results information [30]–but they also seem to be reluctant to copy pure results (see above). The underlying reason might be that a third factor may be responsible for these discrepant findings, and we believe this factor could be social.

In reviewing the ghost condition literature in chimpanzees, we noticed that these studies systematically differed with respect to social factors, which might explain their conflicting findings. Besides actions and results, there is a third–social-type of information that observers may learn about and copy during demonstrations: goal information [10], [11]. Goal information describes the state of the world that the demonstrator tries to achieve. Ghost control studies typically lack such goal information (one cannot gather goals from ‘ghosts’). Recent studies suggest that chimpanzees may be able to perceive more about goals than was previously thought [32], [33]. In the light of such recent findings, it is conceivable that the absence of this type of information may prove detrimental to the observational learning process for chimpanzees, and if that is the case, it is to be expected that chimpanzees' ability to copy will be negatively affected in ghost conditions. In addition to the potential lack of goal information, previous ghost condition studies have also lacked social presence during the demonstration phase. Yet, having a conspecific present during ghost demonstrations may enhance learning by way of social support [34]. Social ghost conditions may act as general motivation enhancers; the only ape study to provide evidence for emulation in a ghost condition found copying only in its *social* ghost condition (i.e., “enhanced ghost condition” [29]). Finally, in those cases where a conspecific is present during demonstrations, it may matter whether there is a separation between observer and demonstrator. For example, in Tennie et al., the demonstrator and observer were separated by a glass/mesh during full model demonstrations, which may have led to this study's negative finding [12]. In both Hopper et al. studies, there was no conspecific present in the ghost condition, and no copying was found there [29], [31]. However, in the Hopper et al. “enhanced” ghost condition (i.e., “ocial”ghost control [29]) a conspecific was present in the same room as the observer (Lydia Hopper, pers. comm.), and it was here that clear evidence for emulation was found in chimpanzees. The reason that little evidence of emulation (i.e., only in the 1st trial) was found even in this “nhanced”condition might also be simply because in this condition the demonstrators did not directly interact with the apparatus, and thus probably did not transmit any goal information. To summarize, it is conceivable that these three social factors play a role in emulation learning in chimpanzees: 1) goal information, 2) the presence of a conspecific during demonstrations, 3) if a conspecific is present, physical proximity between conspecific and observer.

In the present study, we tried to include all of these social factors in our demonstration phases. We also aimed to avoid the potential pitfalls of studies that employ the “two-action task” procedure, especially the problem of triviality of task (e.g. [21]). When using less trivial tasks, one can use two different approaches, the first of which would be to use tasks incapable of being solved by individual chimpanzees (i.e., where low-fidelity learning mechanisms alone do not help). These studies are interesting because they can uncover cases of cumulative culture. Currently, only one such study has been conducted, and the four species of great apes tested failed to show evidence of such copying [5]. A second approach would be to use tasks in which most, but not all, chimpanzees fail during baseline trials–an approach chosen for this study. In these experiments one can use the few successful subjects as “natural demonstrators” since they learned the technique during baseline trials and do not need to be trained further (it should be noted that such a practice may introduce a bias against good inventors. However, this is less problematic as long as several experimental conditions are compared with each other. Here each condition then tests apes with comparable performances). The added benefit of this situation is that the demonstrated technique is potentially more representative of a real ape tradition, and hence more ecologically valid (see [5]; see also discussion). Here, we tested chimpanzee subjects in such a difficult (but not impossible) problem solving task (“floating peanut task”; see [35], [36]). This task consists of a Plexiglas tube mounted vertically to the mesh of a cage, with only the top end open. Shelled peanuts are placed inside the tube resting at the closed bottom. The peanut could not be extracted from the top unless subjects added water to the tube thus causing the peanut to float high enough so that it could then be extracted. Prior to our study, Hanus et al. tested 25 chimpanzees using this task on Ngamba Island, Uganda; as well as 19 chimpanzees at the WKPRC, Leipzig, Germany (total n = 44, [36]). Overall, in Hanus et al. 's study, only 7 subjects were successful, and all of these invented the solution either in their first or their second trial. Thus, in Hanus et al. 's study, subjects either learned early on in the trials or never at all, despite the fact that all subjects received four to eight trials.

We adapted the Hanus et al. study into a social learning experiment in order to ascertain whether chimpanzees are best described as emulators or imitators. All subjects were first tested in a baseline period, in which no previous information was provided to subjects (partly data from Hanus et al. and partly novel baseline trials established by us). Subjects then entered one of two experimental conditions: the full demonstration condition (providing information about actions, goals and results), or the emulation condition (“ater bottle”, providing only information about results and goals). In the full demonstration condition subjects witnessed a model pouring water from the mouth to the tube in order to get access to the peanut. In the emulation condition, subjects were shown how to solve the task by pouring water from a bottle into the tube. Thus, observers were required to produce the alternative, unobserved action (spitting water into the tube) in order to achieve the demonstrated result (i.e., making the peanut float up to the top with water).

By using these three conditions, we set out to disentangle the contributions of different learning processes potentially involved in the floating peanut task–as a model for behavioural traditions in chimpanzees in the field (e.g. [5], [37]). Comparison of the subjects' performances allowed us to do the following: a) measure the probability of innovation in these subjects over trials as a potential general means of solving the problem–and the rate of innovation was determined by baseline performance, b) measure the effects of different demonstration types compared to baseline performance (i.e., whether one or both demonstration types led to more solutions than had occurred during baseline; in other words, whether observational learning could help elicit the behaviour), c) determine the most plausible underlying learning mechanism (imitation or emulation) by comparing the effects of the two demonstration conditions. The underlying logic was that one type of demonstration (the full model; actions, goals and results) would only constitute an advantage if subjects were engaging in action copying (imitation) in order to learn the solutions. However, if no difference between the demonstration conditions could be found the most parsimonious explanation would be that subjects had made use of the same type of information in both conditions (i.e., results information (possibly spurred by goal information)–since this was the only type of information that was present in both experimental conditions).

## Methods

### Ethics statement

All the presented studies were non-invasive and strictly adhered to the legal requirements of the countries in which they were conducted. For Leipzig (Germany), animal husbandry and research complied with the “EAZA Minimum Standards for the Accommodation and Care of Animals in Zoos and Aquaria” and the “WAZA Ethical Guidelines for the Conduct of Research on Animals by Zoos and Aquariums” respectively. For Ngamba Island (Uganda) animal husbandry and research complied with the “PASA Primate Veterinary Healthcare Manual” and the “Chimpanzee Sanctuary & Wildlife Conservation Trust Policy”.

In Leipzig, the apes were housed in semi-natural indoor (overall 533 m^2^ chimpanzee group “A”; overall 340 m^2^ chimpanzee group “B”) and outdoor (4000 m^2^ chimpanzee group “A”; 1400 m^2^ chimpanzee group “B”) enclosures with regular feedings, enrichment and water ad lib. Subjects voluntarily participated in the study and were neither food nor water deprived.

In Ngamba, the apes were allowed to roam freely on the 40 ha island during the day and spent the night in seven interconnected sleeping rooms (overall 140 m^2^) with regular feedings and water ad lib. Subjects voluntarily participated in the study and were neither food nor water deprived.

### Subjects

Thirty-two socially-housed chimpanzees (*Pan troglodytes*) participated in this study. There were eleven males and 21 females, ranging in age between five and 31 years. Twenty-three chimpanzees were housed at the Ngamba Island Chimpanzee Sanctuary (http://www.ngambaisland.org), Uganda and ten were housed at the Wolfgang Köhler Primate Research Center in Leipzig Zoo (http://wkprc.eva.mpg.de), Germany (Table 1). None of the subjects had ever solved this task either because they had never been tested (n = 3, all in Ngamba) or having been tested in a previous study on non-social problem-solving [36], they had failed to solve it. Subjects could choose to stop participating at any time and one subject in Ngamba, “Sophie”, was excluded due to this criterion. After participating, subjects were then released back into their home enclosures.

**Table 1:** Overview of number of trials for baseline experience (newly installed drinker trials only) and type of experimental condition.

Subject	Sex	Housing	Rearing history	Baseline Hanus et al.	Baseline this study	Experimental condition
Alex	M	WKPRC	Hand	*0*	*4*	*Water bottle*
Annett	F	WKPRC	Hand	*0*	*4*	*Water bottle*
Fraukje	F	WKPRC	Hand	*0*	*4*	*Full demonstration*
Gertruida	F	WKPRC	Mother	*0*	*4*	*Water bottle*
Patrick	M	WKPRC	Mother	*0*	*4*	*Full demonstration*
Pia	F	WKPRC	Mother	*0*	*4*	*Full demonstration*
Sandra	F	WKPRC	Mother	*0*	*4*	*Water bottle*
Swela	F	WKPRC	Mother	*0*	*4*	*Full demonstration*
Unyoro	M	WKPRC	Mother	*0*	*4*	*Water bottle*
Asega	M	NICS	Mother/Hand	*8*	*2*	*Water bottle*
Bahati	F	NICS	Mother/Hand	*4*	*0*	*Full demonstration*
Baluku	M	NICS	Mother/Hand	*4*	*0*	*Water bottle*
Becky	F	NICS	Mother/Hand	*8*	*2*	*None*
Bwambale	M	NICS	Mother/Hand	*6*	*0*	*Full demonstration*
Connie	F	NICS	Mother/Hand	*8*	*0*	*Water bottle*
Ikuru	F	NICS	Mother/Hand	*8*	*0*	*Full demonstration*
Indi	M	NICS	Mother/Hand	*8*	*2*	*Full demonstration*
Kalema	M	NICS	Mother/Hand	*8*	*0*	*Water bottle*
Kazahukire	F	NICS	Mother/Hand	*0*	*2*	*Full demonstration*
Kidogo	F	NICS	Mother/Hand	*4*	*2*	*None*
Kisembo	M	NICS	Mother/Hand	*7*	*0*	*Full demonstration*
Nakuu	F	NICS	Mother/Hand	*4*	*0*	*Full demonstration*
Namukiza	F	NICS	Mother/Hand	*4*	*0*	*Full demonstration*
Nani	F	NICS	Mother/Hand	*8*	*0*	*Water bottle*
Natasha	F	NICS	Mother/Hand	*8*	*0*	*Water bottle*
Ndyakira	F	NICS	Mother/Hand	*0*	*2*	*Water bottle*
Nkumwa	F	NICS	Mother/Hand	*8*	*2*	*Water bottle*
Pasa	F	NICS	Mother/Hand	*8*	*0*	*Water bottle*
Sally	F	NICS	Mother/Hand	*8*	*2*	*None*
Sunday	M	NICS	Mother/Hand	*4*	*2*	*None*
Umugenzi	M	NICS	Mother/Hand	*0*	*2*	*Full demonstration*

F = female, M = male; WKPRC = Wolfgang Köhler Primate Research Center; NICS = Ngamba Island Chimpanzee Sanctuary, Uganda. Taken (and extended) from [36]. Included subjects only.

Three additional chimpanzees (two from Ngamba: Yoyo, Umutama and one from Leipzig: Frodo) who had learned to solve the task in a previous study were used during the demonstration conditions. All three individuals were dominant over their partners during the demonstration conditions. This was done to insure that the partners would watch but not interfere with the demonstrations-something that fortunately never happened during the study.

### Materials

A vertically-oriented Plexiglas tube (25 cm long; 5 cm outward diameter and 5 mm thick) closed at the bottom was securely fastened to the caging. One peanut pod (containing two peanuts) was dropped inside the tube so that it rested at its bottom outside of the subject's reach. Prior to testing it was ensured that no tools were available in the cage. A drinker situated within 1 m from the tube (with the spigot at the same height as the tube opening) provided the water source. Such a drinker was installed prior to the test and it was not available outside of the testing situation (such “new drinkers”may protect against functional fixedness potentially attached to “old drinkers” see [36]).

### Procedure

Subjects received two conditions: one baseline condition and one of the two experimental conditions. Prior to receiving one of the experimental conditions, all subjects had received the baseline condition to assess whether subjects were able to solve the task individually. However, subjects differed both in the number of baseline trials that they received, ranging from 2 to 10 (see Table 1) and the source of those trials. In particular, some subjects (included subjects only, see Table 1) received all their baseline trials from the Hanus et al. study (n = 12, see [36]), some only from the current study (n = 12), and some from both studies (n = 7). The reason we conducted our own baseline was to ensure that our baseline and the Hanus et al. ' baseline produced comparable results. We found no differences between the subjects tested with the Hanus et al. baseline and those tested in the current study. Therefore, we pooled all the subjects into the same analysis. The different number of trials was an important feature of our design to be able to assess order effects (see below). Upon completing baseline trials, all subjects except four (due to time constraints) were distributed into two groups matched as closely as possible for age, sex and number of previous trials and received one of the two experimental conditions. Thirteen subjects were placed in the full demonstration condition and 14 subjects were placed in the water bottle condition. Next we describe the baseline and the two experimental conditions.

#### Baseline (total N = 31)

Subjects were presented with the peanut at the bottom of the tube and allowed to attempt to acquire the peanut. The differences with our own baseline trials and those of Hanus et al. 's study were as follows: we let subjects first observe the general setup from an adjacent room (in order to further control for the demonstration/waiting times of our two experimental conditions). Thus, after having observed E place the nuts in the tube, and prior to each trial, subjects spent five minutes in the cage next to the experimental cage (in full view of the tube). Subjects received a maximum of two trials (both on one day) and were alone during trials (except for E, who was present). In this condition, the only way to solve this task was to invent the solution spontaneously.

#### Full model condition (N = 13)

**Figure 1:**
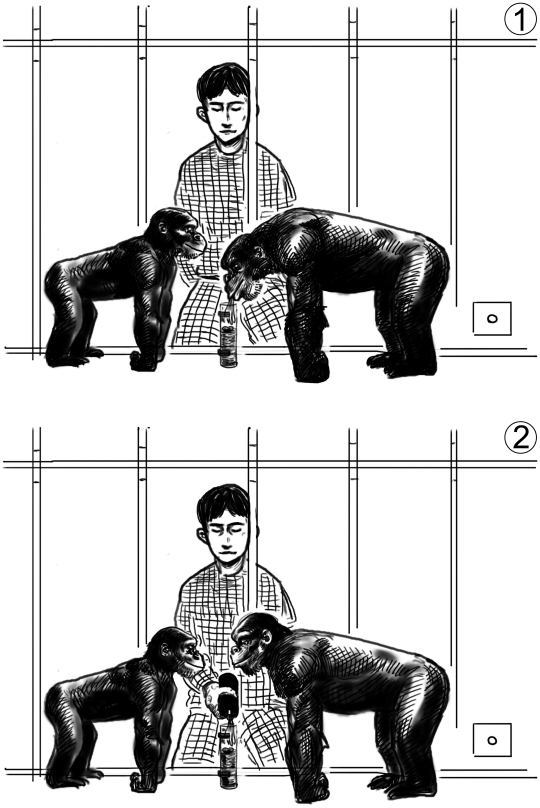
Drawings of the two experimental conditions. 1) full model condition; 2) water bottle condition. Squares in lower right corners represent drinkers. Chimpanzees on the left: subjects. Chimpanzees on the right: demonstrator or stooge (depending on condition). Please note that, for clarity reasons, most bars of the caging have been omitted from the drawing.

This condition was the same as our own baseline (see above) except that prior to their first test trial, subjects witnessed four to six demonstrations of the solution (from the initial water spitting until their partner acquired the peanut), and two further demonstrations before their second trial (see Fig. 1a for the general setup). Subjects received a maximum of two trials, depending on their performance (see below). A conspecific demonstrated the solution (spitting multiple times inside the tube in the process) while the subject stayed in the same cage, which means that she could freely approach and closely observe the demonstrator. Before their first trial, observers were required to have witnessed at least two spits into the tube. If they had seen these two spits within four demonstrations (live coded by E: each time subjects were required to face towards the demonstration, open-eyed and with an unobstructed line of sight), they were given their first trial, if not, they were given two more demonstrations. If observers still had not seen the required two spits, they were excluded from the study (though this situation never arose). In this condition, subjects could invent the solution spontaneously, they could imitate the actions of water spitting (action copying: imitation), or they could only copy the results of the demonstrator's actions.

#### Water bottle condition (N = 14)

This condition was identical to the full model condition (including the number of solutions witnessed) except for the following differences. A solution-naïve, but dominant conspecific (“stooge” demonstrator) was used as a social partner for the subject. E enacted an alternative solution to water-spitting by pouring into the tube the necessary amount of water from a bottle from outside the cage (Fig. 1b). In order to fill the tube to the required level, E poured three ‘glugs’ from the bottle of water. If the observer was constantly watching E, then there was approximately two seconds between each glug. However, if the observer was not watching, then E paused the pouring until the observer was watching, then continued to pour. Once the peanut reached the top of the tube, the ‘stooge’ demonstrator invariably took the peanut, thus being comparable to the outcome of the full demonstration condition. In this condition, the subject was able to witness the results producing the solution (i.e., water added to the tube will raise the water level which will raise the nuts to within reach) but without any actions that the subject could use to solve the task. Subjects in this condition thus had two possible routes to solutions: they could invent the solutions spontaneously, or they could emulate (but not imitate–since they never saw the spitting action).

All trials were terminated after ten minutes unless subjects were still attempting to get the peanut after this period has elapsed. In such case, trials could be extended for a maximum of two additional periods of five minutes so that the maximum length of a trial could be 20 minutes (ten plus five plus five). Trials were also terminated once the subject retrieved the reward. In the event of a success, the subject was not tested again.

### Data Scoring and Analysis

All trials were videotaped using a wide-angle camera. E scored live whether or not a subject was successful in retrieving the nuts in a given trial (i.e., general success was our main dependent measure). Additionally, we scored from the videotapes the following drinker- and tube-related behaviours: number of times water was collected from the drinker and number of times the subject spat into the tube. In order to assess inter-observer reliability for these tube- and drinker-related behaviour a different coder (C. Tennie) coded 20% of trials (randomly selected from all experimental trials, as well as from the baseline trials that were performed solely for this study). Inter-observer reliability was very high for both measurements (Pearson's: number of times water was collected: r = 0.972; number of times spat into tube: r = 0.982). To assess inter-observer reliability for successes a naïve coder also coded general successes from videotape for randomly chosen trials (60% of all trials). Reliability was nearly perfect (with only one mismatch in total).

We analyzed our dependent measure, success to get the peanut, using a generalized linear mixed model (GLMM; [38]) with binomial error structure and logit link function calculated using the package lme4 [39] for R [40]. As fixed effects we included the factor ‘condition’ and the covariate ‘trial number’ into the model. Since the number of successes in the dataset was small, the assumptions of this procedure were likely to have been violated, devaluing the validity of the p-values thus derived. Hence we established correct significances based on a permutation test. For this we randomized the outcomes of trials within subjects and then ran a GLMM for the randomized data. We repeated this procedure 1000 times and each time derived the estimated coefficient of an effect (condition or trial). Finally we estimated the p-value for an effect by determining the proportion of permutations that revealed an absolute coefficient being at least as large as that of the original data.

GLMM offered us two key advantages over other statistical techniques. First, it allowed us to incorporate a “subjects” factor as a random effect in order to control for observations that are replicated [41]. Second, since our baseline always preceded the experimental conditions, this could potentially create an order of administration confound. The inclusion of the covariate ‘trial number’ in the model allowed us to control for this aspect (i.e.: when trial/order effects were tested, then condition was controlled for and vice versa). Thus order/trial effects, if they existed for an experimental condition, would not explain a general effect of condition if it were found.

We used the exact Mann-Whitney-U test to analyze whether there were differences between the two experimental conditions. To do so, we calculated the subjects' success ratios (success divided by total numbers of trials, including baseline trials) and compared the ratios calculated for each demonstration condition. Elsewhere, wherever we used either a Mann-Whitney-U test or a Wilcoxon test we used 1^st^ trial data only (since not every subject had a 2^nd^ trial). Obviously, this rule did not apply when we compared behaviour between the two trials.

To compare drinker- and tube-related behaviour between baseline and experimental conditions, we used our baseline data derived from Ngamba subjects only. Since we could not perform a meaningful Wilcoxon test on just the resulting six subjects who were in both the baseline and the experimental conditions, we ran a Mann-Whitney-U test comparing subjects in the baseline condition with others in the experimental conditions. This procedure was straightforward for those subjects who only were in one condition, but for those subjects who had been in both conditions (i.e., baseline condition and experimental condition) we only used data from their baseline condition (we did so since the sample size of the baseline condition was smaller than the sample size of the experimental condition).

## Results

Overall, eight subjects were successful across the experimental conditions (five in the full model condition and three in the water bottle condition). Both experimental conditions, when compared to baseline, showed significantly more successes after demonstrations (Full model condition; permutation test: p = 0.002; Water bottle condition; permutation test: p = 0.015). We found no additional effects of trials when comparing baseline with both experimental conditions pooled (Effect of exp. condition; permutation test: p = 0.001; Trial effect; permutation test: p = 0.957), When tested alone, the full model condition, but not the water bottle condition, showed additionally an effect of trial (Full model condition; permutation test: p = 0.034; Water bottle condition; permutation test: p = 1.00). Thus, both types of experimental demonstration resulted in more successes than the baseline condition, which means that demonstrations did indeed have a positive effect and thus offered an advantage over individual innovation. While differing in terms of success in retrieving the peanut, baseline subjects did not differ from experimental subjects in tube- and drinker-related behaviour (exact Mann Whitney U tests: number of water retrievals: U = 53, N_BL_ = 10, N_EXP_ = 12, p = 0.673; number of spits into the tube: U = 39.5, N_BL_ = 10, N_EXP_ = 12, p = 0.148).

There were no significant differences between experimental conditions in the success to retrieve the peanut (exact Mann Whitney U test, U = 78, N_full demo_ = 13, N_waterbottle_ = 14, p = 0.475) or the number of subjects who spat into the tube (Fisher's test; p = 0.706; seven and six subjects in the full model and the water bottle conditions, respectively), Furthermore, subjects in both experimental conditions did not differ in general tube-and drinker-related behaviour (exact Mann Whitney U tests: number of water gatherings U = 62.5, N_full demo_ = 13, N_waterbottle_ = 14, p = 0.170; number of spits into tube: U = 79, N_full demo_ = 13, N_waterbottle_ = 14, p = 0.547).

Next we pooled the data from both experimental conditions to explore what might distinguish successful from unsuccessful subjects. Perhaps successful subjects were more motivated to solve the task. If so it would be expected that successful subjects simply tried longer to solve the task than did unsuccessful subjects. Contrary to this idea, we found that successful subjects had shorter trials than unsuccessful subjects (exact Mann Whitney U test, U = 0, N_Success_ = 6, N_NoSuccess_ = 21, p = <0.001). Unsuccessful subjects became less focused on the task in their second trial as evidenced by the fact that they retrieved water less often in their second trial than in their first trial (Wilcoxon; T^+^ = 113.5; n = 16; p = 0.016). Additionally, we found that successful subjects were younger than unsuccessful subjects (exact Mann Whitney U test: U = 21, N_NoSuccess_ = 21, N_Success_ = 6, p = 0.011; median age (years): successful = 7, unsuccessful = 10).

Finally, we checked whether there might have been a difference between the Ngamba and Leipzig subjects concerning drinker- and tube-related behaviour. We detected no such differences (exact Mann Whitney U tests: number of water gatherings: U = 45, N_Ngamba_ = 18, N_Leipzig_ = 9, p = 0.064; number of spits into tube: U = 54, N_Ngamba_ = 18, N_Leipzig_ = 9, p = 0.139).

## Discussion

In stark contrast to baseline performances, both experimental conditions elicited successes in some observers–with no difference between the two experimental conditions. Thus, demonstrations of three simultaneous information types (i.e., actions, goals and results: full demonstration condition) offered no advantage over demonstrations of two information types (i.e., results and goal information only: water bottle condition). The most parsimonious explanation is that the underlying learning mechanism was emulation learning (results copying; here possibly spurred by goal information) in both experimental conditions–since apparently action information offered no advantage to observers. We thus conclude that unsuccessful chimpanzees can be observationally induced to solve the floating peanut task mainly on their own: when trying to arrive at the observed result, they were able to fill in the (unseen) action information themselves.

While this one study alone cannot rule out (spontaneous) action copying in chimpanzees (though see also [30]), our results show that emulation is a viable mechanism for acquiring target behaviour under social circumstances–that is, if presented together with goal information. The idea that some form of emulation could account for tradition-building in chimpanzees is an explanation that is consistent with the ape social learning literature in general ([4], [5], [9], [12], [42], [43] but for a different view see [8]) and with more recent experimental evidence for group-specific traditions forming in monkey species that lack complex imitating abilities [44]. At the moment, the most parsimonious explanation seems to be that copying results and goals (rather than copying of actions) could underlie ape traditions. When social support, spatial separation and goal information are controlled for, chimpanzees showed evidence for copying (of results, i.e., emulation), and with no difference in performance to a full demonstration condition. This finding of copying is in contrast to earlier studies that sought to detect emulation learning in chimpanzees and which presented results information while lacking social controls (i.e. [12], [29], [31]). Importantly, no evidence for copying was found in a study that included these social factors, but which presented no results information at all (“pure” action copying study [30]). It is also worth noting that chimpanzees often do not follow actions demonstrated to them when these same actions are also available to them (e.g. [5], [12])–and instead prefer to act independently from demonstrations, which is further evidence that emulation learning is important for them (see also an example for this in keas: [45]).

Our results are not due to mere stimulus or local enhancement [46], [47] to the drinkers in the full model condition. This information was not necessary, since there was no difference between successes (or indeed any drinker- or tube-related behaviour) elicited by both experimental conditions, despite the fact that there was no drinker enhancement in the water bottle condition (the water bottles were instead filled out of the observer's sight). One might argue that observers would have copied even in cases where water was merely present to some degree in the tube (i.e., either a semi-filled tube, or a fully filled tube)–without having seen the filling of the tube (so called “end-state conditions” [17]). However, we do not think that subjects would have copied in such a stationary condition, for the following reasons: Baseline subjects did not differ from experimental subjects in general drinker- and tube-related behaviour, suggesting that indeed something extra–and crucial for success–has been transmitted by both demonstration types. Also, such semi-end-state conditions (semi-filled tubes at start of trial) were already conducted as part of the problem solving study of Hanus et al. and they found no difference between their fully dry (like in our baseline) and semi-filled condition [36]. In contrast, our dynamic (and social) emulation condition led to successes in subjects who had proven unsuccessful before–which suggests that dynamic physics matter more to chimpanzees than do at least semi-end-states (at least for difficult tasks; for an easier task in chimpanzees with opposite findings see [17]). Or else it may suggest that goal information needs to be additionally present, since this type of information was missing in Hanus et al. [36]. However, the possibility remains that a special end-state condition–a fully filled tube–would be as effective as our emulation condition. Future studies will be needed to address this possibility.

Based on the literature, we believe the following additional factors were ultimately responsible for our finding that chimpanzees are able to invent unseen actions for solutions that they can potentially invent on their own (as evidenced by some successful subjects in Hanus et al. [36]). As hypothesized in the introduction, it is likely to be social factors that lead to clearer evidence for emulation rather than (somewhat non-naturalistic) ghost controls. We aimed to provide observers with as much social information as possible, in order to induce their natural tendency to emulate, and it seems that we have succeeded. What we cannot do, however, is determine which of these three social factors was the most relevant (or whether there was an interaction between them). Chimpanzees in social learning experiments might require only one or else several of the following: goal information, social support and/or non-separation of subjects from observers. Should future studies identify goal information as being strictly necessary for chimpanzees to induce emulation then the learning mechanism itself would require renaming (e.g., teleological emulation: [19]; Else, using the simplified terminology of Call & Carpenter one may speak of “goal and results copying” [10]).

Due to the general ecological validity of our study (in terms of social factors, goal information, conspecific demonstrators [48], as well as using a difficult task), and in light of a previous study that failed to detect action copying in chimpanzees when only action copying would have led to success [30], our finding supports the recent hypothesis that emulation learning via re-invention could, at least in principle, underlie many, most, or all socially learnt behaviours in wild chimpanzees [5]. Once one subject has found the required solution, it will be considerably easier for others who observe her to derive at the (same) solution themselves (as shown in this study). In accordance with this view, there seems to be no behavioural tradition in chimpanzees (or any great ape-) which could not be invented by a single (perhaps specially gifted, or perhaps especially “lucky” or motivated) individual–and then spread by way of emulation learning (possibly helped by enhancement effects). It is apparently unnecessary for actions to be copied during such a process–emulation suffices. It is true that not all observers in our study acquired the target behaviour (i.e. successful behaviour), suggesting that additional factors might be necessary before a behaviour appears on a population-wide scale (e.g. more demonstrations or equal levels of motivation etc.; but see also below for a hypothesis based on age-effects).

By emulation, observers in effect “re-invent” a solution once they have witnessed it–an effect best described as “catalystic”, rather than as “transmissive” (i.e., a domino-like effect). This would mean that great apes like chimpanzees can only learn what they could, in principle, also invent on their own–at least given the right individual circumstances (i.e., enough motivation, access to all necessary material, focus on the right objects, reduced neophobia, social support etc.). The sheer number of these interacting factors ensures that, overall, such inventions (and re-inventions) must be regarded as a probabilistic process, and so, while the appearance of certain behaviours in a given single chimpanzee can still have a low baseline probability, the fact remains that the task could potentially be learned in its entirety without the help of observational learning at all (example of such “atent solutions”include: chimpanzee nutcracking (see one subject in the baseline of a recent study [49], which may have invented this solution spontaneously); gorilla nettle feeding: [42], [43]; chimpanzee leaf swallowing: [50]; chimpanzee termite fishing: [51]; capuchin nut-cracking: [52]). During the spread of the behaviour, the necessary actions then are generated from within each observer anew and independently–and thus actions not copied (and, crucially, they do not need to be copied).

This view has implications for the general limits of ape traditions. If this hypothesis [5] proves correct, then ape traditions consist entirely of “atent solutions” the scope of which is basically determined by the limits of the emulative capacities of the species (in other words: by the underlying problem solving skills–developed via natural selection). Additionally, many other factors likely play a role in the realization of traditions (e.g. motivational differences between populations due to prior food choices). In concert, these factors may lead to the observed “patchy pattern” of traditions across living populations of chimpanzees (e.g., they lead to different “atent solution”mixtures in different populations, which explains the mosaic picture of chimpanzee traditions described by Whiten et al. [37]–for which human like imitative abilities are usually claimed as the underlying reason).

Our findings confirm an earlier observational learning study [53] that described a similar age effect in emulation in chimpanzees–and that also used a difficult, but not impossible, task [compare also 5]. Noting that the age of successful learners (4–6 years) coincided with the “earliest tool-use behaviours in the wild” Tomasello et al. [53] introduced a “critical time period” hypothesis. Thus, the reason why most (or all) chimpanzees in a given wild population show skill in certain tool-“traditions”–in contrast to our and others' [53] more partial findings–might be that, earlier, these chimpanzees were able to learn during their critical time period. If true, this hypothesis would explain why not all subjects in our and the other [53] study became successful after demonstrations. Once subjects have become too old they might cease to be able (or to be motivated) to emulate in such situations.
